# Phenotypic characterization of heifer development using carcass ultrasound in Angus cattle

**DOI:** 10.1093/jas/skag045

**Published:** 2026-02-16

**Authors:** Sarah L Phelps, Garland R Dahlke, Jaedyn M Condon, Patrick B Wall, Miranda M Culbertson

**Affiliations:** Department of Animal Science, Iowa State University, Ames, IA 50011, United States; Department of Animal Science, Iowa State University, Ames, IA 50011, United States; Department of Animal Science, Iowa State University, Ames, IA 50011, United States; Department of Animal Science, Iowa State University, Ames, IA 50011, United States; Department of Animal Science, Iowa State University, Ames, IA 50011, United States

**Keywords:** beef cattle, carcass ultrasound, heifer pregnancy

## Abstract

The utility of carcass ultrasound measurements in heifer development as indicators of reproductive performance was evaluated. Current methods of assessing heifer development include body weight (BW), nutritional status, and body condition score. However, these methods do not measure actual body composition, and the relationship between actual pre-breeding body composition measurements and heifer fertility is poorly understood. Measurements were collected on 124 purebred Angus heifers at the Iowa State University McNay Memorial and Demonstration Research Farm. One week post-weaning (day 0), heifers were randomly allocated into two dietary treatment groups stratified by age and weight. One group of heifers was developed to reach 55% of expected mature BW at the time of breeding (restricted), and the second group was developed to reach 65% of expected mature BW at breeding (non-restricted). Carcass ultrasound measurements and BW data were collected at six time points during heifer development from October 2023 to September 2024 (day 0, 55, 123, 178, 231, and 338). Carcass ultrasound measurements included ribeye area (REA), backfat thickness (FAT), rump fat thickness (RUMP), and percent intramuscular fat (IMF). A controlled internal drug release (CIDR) was used to synchronize heifer estrus cycles for timed-artificial insemination (AI), and Estrotect heat detection patches (Estrotect, Rockway, Inc., Spring Valley, WI) were used to measure estrus response. The final pregnancy diagnosis was made by a veterinarian on day 338, 12 weeks post initial AI in September 2024. Data were analyzed using R Statistical Software 4.4.1. Treatment group differences (*P* < 0.05) were identified at day 55 for mean measurements of weight, REA, FAT, and RUMP, and at day 178 for IMF. Estrous response rate was higher in the non-restricted group than the restricted (*P* < 0.05), but the overall pregnancy rates between groups was not significant (*P* = 0.79). Two logistic regression models were analyzed to capture pregnancy response using body composition measures. Backfat was a significant predictor of heifer pregnancy (*P* < 0.05). Results from this study can help beef cattle producers determine the appropriate percentage of expected mature BW for developing heifers in their operation, based on specific management practices. Additionally, findings can inspire prospective researchers to further investigate the ideal FAT levels for optimal heifer reproductive performance.

## Teaser

Different heifer developmental programs influence the growth of body composition traits and subsequent heifer reproduction.

## Introduction

Calf sales are the primary revenue source for beef cow-calf systems, making the reproductive success of females in the herd an influential factor for determining the profitability of an operation. Heifers that conceive and calve early in the calving season continue to calve early in subsequent seasons and stay longer in the herd (Lesmeister et al. 1973). A reproductively efficient heifer will reach puberty around 12–13 months and conceive by 15 months of age (Byerley et al. 1987; Patterson et al. 1992; Roberts et al. 2019). Heifers that fail to reach puberty during the breeding season result in a reproductive failure (Lesmeister et al. 1973; Whittier et al. 2008); therefore, heifer development practices should focus on facilitating puberty onset.

Higher body weight (BW) and nutritional status have been shown to decrease the age at puberty. Heavier heifers fed a higher average daily gain diet tend to reach puberty earlier than heifers at a lighter weight fed a lower gain diet (Wiltbank et al. 1969; Hall et al. 1995; Gasser et al. 2006; Day 2015). Recommendations suggest heifers should reach 60–65% of expected mature BW at the time of breeding. Research has demonstrated that developing heifers to 50–55% of expected mature BW may be more economically efficient than developing heifers to larger BWs (Funston and Larson 2011). Aside from BW, other commonly used indicators include body condition scoring (BCS) to measure nutritional status and reproductive tract scoring (RTS) for assessing reproductive maturity. Scores for RTS range from 1 to 5, with a score of 1 indicating an immature, non-cyclical tract and a score of 5 indicating a mature, cyclical tract with the presence of a corpus luteum (Andersen et al. 1991). In general, heifers should score a RTS of 4 or 5, as this indicates a cyclical tract, which has been shown to increase pregnancy outcome (Dickinson et al. 2019). Scores for BCS range from 1 to 9, with scores of 5 or 6 being ideal prior to breeding (Thomas and Bailey 2021). This ensures the female has adequate nutrients to initiate pregnancy (Short and Adams 1988). Scores below a 5 indicate underconditioned females, and scores above a 6 indicate over conditioned females, both of which have been associated with decreased fertility (Wettemann et al. 2003; Martínez et al. 2021).

Issues with BCS include the subjectivity and variability of the assigned score based on the individual’s previous experience assigning scores (Vizcarra and Wettemann 1996). Since BCS is scored visually, factors, such as hair coverage, frame score, and body length are all factors that can skew scores (Ferreira and Vedovatto 1996; Thomas and Bailey 2021). Carcass ultrasound imaging provides a potential approach to more accurately measure these traits, as it has been shown to be a reliable estimator of actual body composition (Greiner et al. 2003). Moderate to high genetic correlations between carcass ultrasound measurements and actual carcass traits have contributed to the genetic improvement in ribeye area, backfat thickness, and intramuscular fat. Previous studies have estimated genetic correlations between heifer pregnancy and carcass ultrasound traits to be low and positive, indicating that selection for improvement in both traits can occur (Boldt et al. 2018). The objectives of this study were to: 1) explore body composition trait development in growing heifers, 2) identify how different management programs can influence muscle and fat deposition, and 3) determine the association between heifer pregnancy and body composition traits. We hypothesize that developing heifers to different BWs at breeding will affect body composition measurements taken throughout development and ultimately influence pregnancy status.

## Materials and methods

### Animal care

All methods and procedures used in this research study were approved by the Iowa State University Institutional Animal Care and Use Committee.

### Animal population

Data collected on 124 purebred Angus heifers from the Iowa State McNay Memorial Research and Demonstration Farm located in Chariton, Iowa, were used in this study. These heifers were sired from 19 different sires, and all dams originated from the McNay herd. Minimizing the inbreeding of this herd has been a consistent priority over the years; therefore, a high level of genetic relatedness is not a major concern. No additional feed supplement was provided to heifers during pre-weaning development. At the start of the study, the average mature weight of the McNay cow herd was 615 kg, which was used as the basis for formulating the diets.

Initial measurements for BW and carcass ultrasound traits were collected one week post-weaning (day 0) and continued through final pregnancy diagnosis, assigned by a veterinarian (day 338), 30 days following the end of the breeding period. Heifers were not restricted from feed or water before weigh days, and both groups of heifers were weighed on the same days. At day 0, heifers were allocated into two dietary groups using stratified randomization based on age and weight: a lower average daily gain (restricted; n = 61) and a moderate average daily gain group (non-restricted; n = 63). Unequal group numbers resulted from the culling of two heifers from the restricted group on day 55 due to health reasons, reducing the total number in the restricted group from 63 to 61. The mean age of heifers on day 0 in both groups was 185 ± 18 days, and the mean weight for the restricted and non-restricted groups was 195.2 ± 26.1 and 198.4 ± 25.2 kg, respectively. Table 1 presents the diets fed to each group on a group basis up to AI breeding.

**Table 1:** Treatment group diets for developing heifers from one-week post-weaning (day 0) until artificial insemination (day 231).

	Day 0 to 178^1^		Day 178 to 231^1^	
	Restricted^2^	Non-restricted^3^	Restricted^2^	Non-restricted^3^
**Ingredient, % dry matter basis**				
**Ground hay**	43.9	22.9	71.2	38.0
**Modified corn distillers**	12.0	21.8	9.8	19.7
**Corn silage**	42.5	54.5	17.8	41.3
**Grower mineral**	1.6	0.8	1.2	1.0
**Analyzed composition**				
**NE_m_, Mcal/kg**	1.4	1.8	1.1	1.6
**NE_g_, Mcal/kg**	0.8	1.1	0.5	1.0
**Crude protein, %**	10.8	13.6	10.3	14.1
**Programmed gain^4^**				
**Daily gain, kg**	0.4	0.6	0.3	0.9
**Gain: feed**	0.1	0.1	0.1	0.1
**Actual gain^5^**				
**Average daily gain, kg**	0.7	1.0	0.3	0.3

1 On day 178, diets were modified to prevent the overdevelopment of both heifer groups at breeding.

2 Restricted: diet to develop heifers to approximately 55% of expected mature body weight at time of breeding.

3 Non-restricted: diet to develop heifers to approximately 65% of expected mature body weight at time of breeding.

4 Programmed gain: targeted gain resulting from the formulated diet.

5 Average actual gain, calculated as the average total weight gain over days on feed during each period.

Both treatment diets consisted of ground hay, modified distillers, corn silage, and grower mineral. However, the diet of the restricted group included lower amounts of modified distillers and corn silage and higher amounts of ground hay and grower mineral to achieve an expected mature BW at breeding compared to the diet formulated for the non-restricted group. The treatment diets were developed for heifers in the restricted group to achieve 55% of expected mature BW and heifers in the non-restricted group to reach 65% of expected mature BW at the time of breeding. At the third measurement (day 123), heifers in both groups were predicted to be overdeveloped at breeding. Therefore, the diet was adjusted for both groups starting on day 178 to reduce the ADG to meet the target BW.

### Data collection

Measurements were collected over eight time points between October 2023 and September 2024. Table 2 denotes the month and year of collection points and the measurements collected. Initial measurements for carcass ultrasound traits and BW were collected one-week post-weaning (day 0) and continued to be collected approximately every 60 days through estrus synchronization (day 231). Ultrasound carcass imaging was performed by one consistent certified ultrasound technician throughout the study using Evo II (IBEX® Evo II®, E.I. Medical Imaging, 170 Loveland, CO), and images were analyzed by The CUP Lab® in Ames, Iowa. Carcass images included ribeye area (REA; cm^2^), backfat thickness at the 12th rib (FAT; mm), intramuscular fat percentage (IMF; %), and rump fat thickness (RUMP; mm).

**Table 2:** Measurements collected at each time point between October 2023 and September 2024.

Measurement	Day 0	Day 55	Day 123	Day 178	Day 182	Day 231	Day 241	Day 274	Day 338
**Body weight**	X	X	X	X		X	X	X	X
**Carcass ultrasound^1^**	X	X	X	X		X			X
**RTS and PA^2^**					X				
**Exposure response^3^**							X		
**Pregnancy diagnosis^4^**								X	X

1 Rump fat thickness was not collected on Day 0 due to the small body size of the heifers.

2 Reproductive tract score (RTS) and pelvic area (PA) measurements.

3 Exposure response to estrous synchronization measured in abrasion of Estrotect heat patch.

4 Pregnancy diagnosis assigned as bred by artificial insemination, natural service, or not bred (open).

Fifty-nine days before breeding on day 182, pelvic area measurements and reproductive tract scores (RTS) were taken by a veterinarian. The pelvic area was determined using a pelvimeter (Rice pelvimeter; Lane Manufacturing, Denver, CO) by multiplying the height between the product sacral vertebrae and the symphysis pubic, and the width between the ileal shafts (Patterson and Herring 2022). Eleven heifers did not meet farm management criteria for pelvic area and RTS and were subsequently culled and removed from the study. In selected heifers, estrus was synchronized for artificial insemination (AI) using the 7-day CO-Synch + CIDR protocol. Gonadotropin-releasing hormone (GnRH) was administered, and a controlled internal drug release, or CIDR (CIDR®, Zoetis, Parsippany, NJ, USA), was inserted in the last week of May (day 231). Seven days following insertion, the CIDR was removed, a prostaglandin injection was administered, and an Estrotect heat detection patch was placed on the tailhead of heifers to monitor estrus response (Estrotect, Rockway, Inc., Spring Valley, WI). At breeding, the percent abrasion of each heat patch was recorded, and patch scores (PS) were assigned based on abrasions. Following previous work, PS were assigned based on the percentage of the heat patch that was abraded: 1 for 0–35%, 2 for 35–65%, and 3 for greater than 65% (Walker et al. 2014). Heifers that expressed estrus before timed AI breeding were bred 12 h following expression. All other heifers were bred at timed AI, 54 h after CIDR removal, and administered a GnRH injection. Four different technicians participated in the AI breeding of heifers.

After AI breeding, heifers were managed as one group on pasture without additional supplementation. Bulls were released with heifers at a 1:20 bull-to-heifer ratio, 10 d following AI for natural service (NS). Four weeks post-AI, pregnancy status was determined via transrectal ultrasound to determine AI-bred heifers. Bulls were removed from heifers in August after an 8-wk breeding season. The final pregnancy diagnosis was conducted by a veterinarian 12 wk after AI breeding and 30 days following bull removal in September (day 338). Heifers were designated as not pregnant (open), AI-bred, or NS-bred upon final pregnancy diagnosis.

### Statistical analysis

Data analysis was performed using R version 4.4.1 (R Core Team 2024). Treatment group mean differences in carcass ultrasound measurements, BW, and pelvic area were evaluated using independent two-sample t-tests and a significance level of *P *< 0.05. A Pearson’s Chi-squared test with Yates’ continuity correction was performed to identify if a statistically significant difference was present between heifers that scored a PS of 1 and heifers that scored a PS of 2 or 3 between the two treatment groups. A Wilcoxon rank-sum test was performed to evaluate differences on RTS between treatment groups due to violation of normality and homoscedasticity assumptions for RTS and estrus response data. Pregnancy rates between both treatment groups were compared with a chi-square test of independence.

Two logistic regression models were evaluated to determine the association between heifer pregnancy and carcass ultrasound measurements. Pregnancy response was evaluated as a binary response (pregnant or open) and as a multinomial response (AI, NS, or open). The first model (M1) evaluated the binary response and was fit using the glm function in R (R Core Team 2024) with pregnancy outcome as a binary response of pregnant or not pregnant. The second model (M2) evaluated the multinomial response and was a multinomial logistic regression performed using the multinom from the nnet package in R (R Core Team 2024) with three levels of pregnancy response: AI, NS, and open. Both models initially included treatment group, weight, age, and carcass measurements taken at day 231 for FAT, REA, and IMF as fixed effects. Due to high correlation (r = 0.82) with FAT, RUMP was omitted from the initial model. Stepwise Akaike Information Criterion (AIC) was used to identify the best predictors in each model. Upon identifying the best-fit model, analysis of variance (ANOVA) type III was used to identify significant predictors in each model.

The best-fit logistic regression model for M1is as denoted below:


$$\ln(\frac{P(Y_{i}=\mathrm{pregnant})}{P(Y_{i}=\mathrm{Open})})=\beta_{0}+\beta_{1}X_{\mathrm{Group}}+\beta_{2}X_{\mathrm{FAT}}+\beta_{3}X_{\mathrm{IMF}}$$

Where P expresses probability and Y is defined as the pregnancy response for individual i. In contrast, the probability of an unsuccessful pregnancy for individual i can be denoted as $Q_{i}={1-P}_{i}.$ The regression coefficient, represented by $\beta_{k}$, where k = 1 denotes the group effect coefficient, k = 2 corresponds to the FAT effect coefficient, and k = 3 is the IMF effect coefficient. Predictor variables are represented by X for treatment group, FAT, and IMF. Treatment group was fit as a categorical variable coded as 1 or 2; FAT and IMF were fit as covariates. The intercept term is represented as $\beta_{0}$.

The best-fit multinomial logistic regression for M2 is represented by the following:


$$ln(\frac{P(Y_{i}=AI)}{P(Y_{i}=O)})=\beta_{0}+\beta_{1}X_{\mathrm{Group}}+\beta_{2}X_{\mathrm{FAT}}$$

$$ln(\frac{P(Y_{i}=NS)}{P(Y_{i}=O)})=\beta_{0}+\beta_{1}X_{\mathrm{Group}}+\beta_{2}X_{\mathrm{FAT}}$$

Where equation 2 represents the probability of AI pregnancy compared to being open, and equation 3 models the probability of NS pregnancy compared to being open. The intercept term is denoted by $\beta_{0}$, and the regression coefficients are represented by $\beta_{1}$ in both equations. Treatment group and FAT were fit as predictor variables represented by X, with group fit as a categorical variable, and FAT was fit as a covariate.

## Results

### Pre-breeding development

Mean values and standard deviations for pre-breeding measurements, split by treatment group, are reported in Table 3. On average, the non-restricted group had higher values than the restricted group for all measurements taken between day 55 and 231, with the exception of IMF. Significant (*P* < 0.05) differences in treatment group means for weight, FAT, RUMP, and REA were observed on days 55, 123, 178, and 231. On days 178 and 231, significant (*P *< 0.05) differences in treatment group mean IMF were observed.

**Table 3:** Group means ± SD for observations of body weight (BW) and carcass ultrasound measurements for backfat thickness (FAT), ribeye area (REA), and intramuscular fat (IMF) for Angus beef heifers from one-week post-weaning (day 0) to AI breeding (day 231) when developed to different percentages of expected mature weight.

Day	BW, kg	FAT, mm	REA, cm²	IMF, %	RUMP, mm^1^
**Restricted group (*n *= 61)^2^**					
**0**	195.25 ± 26.06	2.33 ± 0.84	42.86 ± 6.07	3.79 ± 0.95	–
**55**	234.77 ± 29.20*	3.24 ± 0.99*	46.39 ± 6.09*	4.10 ± 0.99	3.53 ± 1.09*
**123**	282.73 ± 33.09*	3.47 ± 1.21*	55.77 ± 6.49*	4.54 ± 1.33	3.71 ± 1.24*
**178**	326.23 ± 32.60*	4.41 ± 1.28*	61.26 ± 6.83*	4.89 ± 1.47*	4.61 ± 1.33*
**231**	339.68 ± 33.90*	3.46 ± 1.40*	60.44 ± 6.70*	4.43 ± 1.38*	4.33 ± 1.58*
**Non-restricted group (*n* = 63)^3^**					
**0**	197.61 ± 24.82	2.19 ± 0.81	43.27 ± 5.80	3.57 ± 0.77	–
**55**	243.51 ± 26.43*	3.60 ± 1.09*	50.75 ± 6.43*	3.86 ± 0.79	4.01 ± 1.31*
**123**	312.72 ± 30.33*	4.59 ± 1.42*	63.56 ± 7.22*	4.75 ± 1.10	5.43 ± 1.60*
**178**	378.26 ± 30.17*	6.90 ± 1.41*	68.99 ± 7.71*	6.02 ± 1.63*	7.24 ± 1.47*
**231**	396.62 ± 30.95*	6.58 ± 1.82*	72.42 ± 7.38*	5.83 ± 1.60*	7.59 ± 1.74*

1 Rump fat thickness was not collected on day 0 due to the small body size of heifers, but was collected for each subsequent date.

2 Restricted: Heifers develop to approximately 55% of expected mature body weight at the time of breeding.

3 Non-restricted: Heifers develop to approximately 65% of expected mature body weight at the time of breeding.

* *P*-value < 0.05: Represents a significant difference in mean measurement values between the restricted and non-restricted group

To quantify the degree of re-ranking of animals between consecutive measurement points (ie day 0–55, 55–123, 123–178, and 178–231; Table 4), Spearman rank correlation coefficients were calculated within treatment group. A high Spearman rank correlation coefficient indicates that the rank of animals for a specific trait, relative to treatment group cohorts, did not change considerably from one collection point to the next. This would suggest the rate of development is monotonic between heifers of the same treatment group during a given timeframe. A low Spearman rank correlation indicates the rank of animals within treatment group for a specific trait changed considerably between two timepoints. This could indicate that a considerable amount of growth and development is still occurring for a specific trait during a given period. All Spearman rank correlation coefficients were different from zero (*P *< 0.05). For most measurements, the non-restricted group initially had lower rank correlations than the restricted group, except for IMF. Low correlations for IMF of 0.32 in the restricted group and 0.52 in the non-restricted group were observed between day 0 and 55. Subsequent rank correlations increased with time for both treatment groups. This would suggest a considerable amount of development for IMF as measured by ultrasound occurred between day 0 and day 55 for both treatment groups, but this amount of development decreased as the heifers became older. All Spearman rank correlations for BW and REA are high, suggesting a monotonic relationship between the heifers within a treatment group for these traits throughout pre-breeding development. The lowest rank correlations for BW and REA in both groups were between days 178 and 231, with correlations for BW of 0.83 in the restricted group and 0.87 in the non-restricted group, and correlations for REA of 0.82 in the restricted group and 0.80 in the non-restricted group. For FAT and RUMP, rank correlations were slightly lower than those rank correlations for BW and REA during the same periods. This would suggest there were slightly more developmental changes for FAT and RUMP compared to REA and BW during pre-breeding development. The lowest correlation for FAT in the restricted group was between day 178 and day 231, with a correlation of 0.73. In the non-restricted group, the lowest correlation was between day 0 and day 55, with a correlation of 0.60. Correlations for RUMP were lowest in both treatment groups between day 55 and day 123 and increased with time.

**Table 4:** Spearman rank correlations for body weight BW, ribeye area (REA), backfat thickness (FAT), and intramuscular fat (IMF) measured within Angus heifer groups prior to breeding at day 231. Correlations are calculated between consecutive measurement days (day 0–55, 55–123, 123–178, and 178–231).

Day	BW	REA	FAT	RUMP	IMF
**Restricted (*n *= 61)^1^**					
**0–55**	0.96*	0.88*	0.79*	–	0.32*
**55–123**	0.97*	0.85*	0.74*	0.79*	0.63*
**123–178**	0.90*	0.83*	0.80*	0.79*	0.73*
**178–231**	0.83*	0.82*	0.73*	0.88*	0.82*
**Non-restricted (*n* = 63)^2^**					
**0–55**	0.89*	0.92*	0.60*	–	0.52*
**55–123**	0.92*	0.86*	0.78*	0.64*	0.68*
**123–178**	0.94*	0.81*	0.78*	0.65*	0.72*
**178–231**	0.87*	0.80*	0.80*	0.79*	0.81*

1 Restricted: Heifers develop to approximately 55% of expected mature body weight at the time of breeding.

2 Non-restricted: heifers develop to approximately 65% of mature body weight at the time of breeding.

* *P*-value < 0.05: Spearman rank correlation coefficient significantly differs from zero.

**Figure 1:**
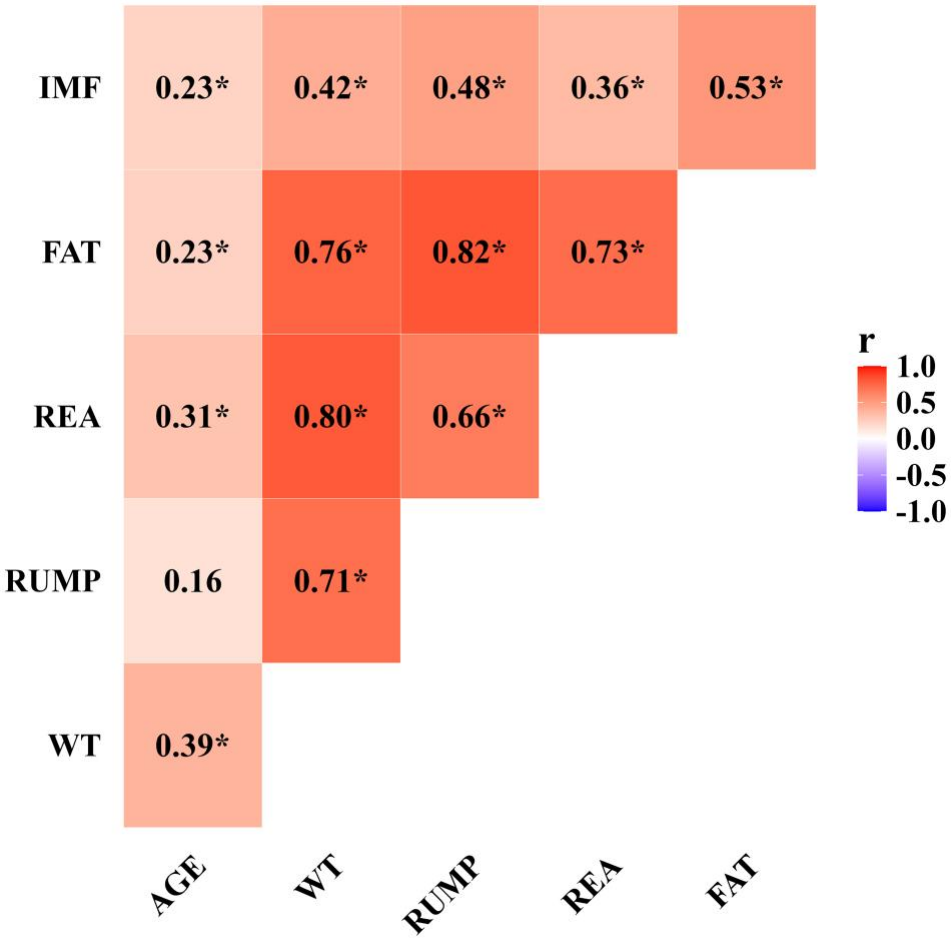
Pearson’s correlation coefficients (*r*) between age, body weight, and carcass ultrasound measurements for (WT), rump fat (RUMP), ribeye area (REA), backfat thickness (FAT), and intramuscular fat (IMF) in developing Angus heifers at the time of breeding. Pearson’s correlation coefficients that significantly (*P* < 0.05) differ from zero are denoted by an asterisk (*).

Pearson’s correlation coefficients (Figure 1) were estimated between age, BW, and carcass ultrasound measurements collected on day 231. All correlation coefficients were different from zero (*P* < 0.05), except for the correlation between RUMP and age (*P* = 0.08). Moderate to strong positive correlations, ranging from 0.66 to 0.82, were identified between all measurement pairs for BW, RUMP, REA, and FAT. This indicates that heifers with high values for one of these traits are likely to have high values for the other three traits. Weak to moderate positive correlations ranging from 0.23 to 0.53 were identified between all measurements with IMF. Finally, low to moderate correlations ranging from 0.16 to 0.39 were observed between age and all traits. This suggests that carcass trait performance and pre-breeding BW in a group could be more strongly influenced by factors other than age.

Treatment group measurements for RTS and pelvic area collected prior to AI breeding are presented in Table 5. The restricted group had a lower mean RTS than the non-restricted group, but the difference was not significant (*P* = 0.15). Mean pelvic area measurements were different between groups (*P* < 0.05). Lower for the restricted group compared to the non-restricted group. Heifers with small pelvic areas and low RTS were culled prior to AI breeding, resulting in seven heifers from the restricted group and four from the non-restricted group being removed from the study.

**Table 5:** Pre-breeding mean ± SD measurements for reproductive tract score (RTS) and pelvic area for heifers from different treatment groups (restricted vs non-restricted).

Measurement	Restricted^1^	Non-restricted^2^
***N***	61	63
**RTS**	3.75 ± 1.29	4.15 ± 1.10
**Pelvic area**	162.24 ± 16.38*	174.09 ± 17.56*

1 Restricted: heifers developed to approximately 55% of expected mature body weight at the time of breeding.

2 Non-restricted: heifers developed to approximately 65% of expected mature body weight at the time of breeding.

* *P*-value < 0.05: Represents a significant difference in mean measurement values between the restricted and non-restricted group.

Estrous response was recorded on day 241 and was analyzed as a percent abrasion of Estrotect heat patch, and a corresponding PS of 1, 2, or 3 was assigned (Table 6). In practice, heat detection aids measure mounting behavior and serve as a visual indicator of estrus. Higher levels of abrasion indicate more mounting behavior and suggest that a female is in standing estrus and receptive to breeding, while lower levels of abrasion may indicate she is in the early stages of estrus or not in estrus. At time of AI, there were no heifers missing an Estrotech heat patch, and as a result, all heifers received an observation for estrus response. Following previous work by Walker et al. (2014), the number of PS 1 observations was compared to the combined number of PS 2 and 3 observations. A Pearson’s chi-square test with Yates’ continuity correction revealed that the proportion of heifers with a PS of 2 or 3 compared with heifers that scored a PS of 1 differed significantly *(P* < 0.05) between the two groups. In the restricted group, 27 heifers scored a PS of 1, and 34 heifers scored a PS of 2 or 3. In the non-restricted group, 8 heifers scored a PS of 1, while 34 heifers scored a PS of 2 or 3.

**Table 6:** Total number of patch scores (PS) assigned by group at the time of artificial insemination (AI).

Measurement	Restricted^1^	Non-restricted^2^
**N**	53	59
**PS 1^3^**	27*	8*
**PS 2 and 3^4^**	34*	34*

Estrous response was measured by the percentage of Estrotect heat patch abrasion.

1 Restricted: heifers developed to approximately 55% of expected mature body weight at the time of breeding.

2 Non-restricted: heifers developed to approximately 65% of expected mature body weight at the time of breeding.

3 Patch Score 1: No abrasions or less than 35% abrasion of heat patch.

4 Patch Score 2 and 3: PS 2, between 35% and 65% abrasion of heat patch and PS 3, greater than 65% abrasion.

* *P*-value < 0.05 represents a significant difference in the proportion of patch scores between heifers in the restricted group and heifers in the non-restricted group at the time of artificial insemination (AI).

### Post-AI performance

Mean treatment group measurements and standard deviations from measurements collected before breeding (day 231) and at final pregnancy diagnosis (day 338) are presented in Table 7. Significant (*P* < 0.05) differences in treatment group means for all traits were found on day 231 and day 338. On average, both groups lost REA, FAT, and RUMP while on pasture management, with the magnitude of loss being larger for the non-restricted group. An increase in IMF was observed in both groups during this period. On average, heifers in the restricted group continued to gain BW on pasture management, while heifers in the non-restricted group lost BW.

**Table 7:** Mean ± SD for body weight (BW) and carcass ultrasound measurements for backfat thickness (FAT), ribeye area (REA), and intramuscular fat (IMF) collected at artificial insemination (AI) breeding (day 231) and final pregnancy determination (day 338) in heifers.

Measurement	Treatment group	Day 231	Day 338
**BW, kg**	**Restricted^1^**	339.68 ± 33.90*	361.36 ± 38.61*
	**Non-restricted^2^**	396.62 ± 30.95*	390.90 ± 30.16*
**FAT, mm**	**Restricted**	3.46 ± 1.40*	3.29 ± 1.31*
	**Non-restricted^2^**	6.58 ± 1.82*	4.12 ± 1.41*
**REA, cm²**	**Restricted^1^**	60.44 ± 6.70*	55.94 ± 6.90*
	**Non-restricted^2^**	70.43 ± 7.38*	60.04 ± 6.79*
**IMF, %**	**Restricted^1^**	4.43 ± 1.38*	5.99 ± 1.98*
	**Non-restricted^2^**	5.83 ± 1.60*	7.02 ± 1.95*
**RUMP, mm**	**Restricted^1^**	4.33 ± 1.58*	3.76 ± 1.27*
	**Non-restricted^2^**	7.59 ± 1.74*	4.96 ± 1.41*

All mean values for traits were significantly different (*P* < 0.05) between the restricted and non-restricted group on day 231 and day 338.

1 Restricted: Heifers developed to 55% of expected mature BW at the time of breeding.

2 Non-restricted: Heifers developed to 65% of expected mature BW at the time of breeding.

* *P*-value < 0.05: Represents a significant difference in mean measurement values between the restricted and non-restricted group.

### Reproductive performance

The results from the initial AI pregnancy determination collected on day 274 and the final pregnancy diagnosis collected on day 338 are presented in Table 8. The discrepancy in animal numbers at the time of pregnancy diagnosis was due to a missing heifer observation in the restricted group at AI pregnancy determination. This heifer was first noted to be missing at AI pregnancy determination and was later found in a pasture containing mature cows belonging to the ISU McNay Memorial and Demonstration Farm. After being located, she was returned to her group of contemporaries.

**Table 8:** Pregnancy rates for heifers 30 days following artificial insemination (AI) and 30 days following bull removal.

	Pregnancy rates 30 days following AI		Final pregnancy rates 30 days following bull removal	
	Restricted^1^	Non-restricted^2^	Restricted^1^	Non-restricted^2^
**N**	52	59	53	59
**AI**	38.54%	49.15%	30.19%	45.76%
**NS^3^**	NA	NA	54.72%	37.29%
**Open**	61.46%	50.85%	15.09%	16.95%
**Total pregnant**	38.54%	49.15%	84.91%	83.05%

Pregnancy rates were categorized as AI bred (AI), bull bred or natural service (NS), and not pregnant (Open).

1 Restricted: Heifers developed to 55% of expected mature body weight at the time of breeding.

2 Non-restricted: Heifers developed to 65% of expected mature body weight at the time of breeding.

3 NS bred could not be determined at initial pregnancy determination.

Pregnancy determination 30 days post-AI found heifers bred by AI was 10.61% higher for the non-restricted group compared to the restricted. However, no statistically significant (*P* = 0.14) association was found between pregnancy status (AI or open) due to treatment group. The final pregnancy percentage was 1.85% higher in the restricted group compared to the non-restricted group, and this difference was also not significant (*P* = 0.76). Pregnancy percentages for AI-bred decreased in both groups between initial determination and final diagnosis, with two pregnancy losses in the non-restricted group and four in the restricted group.

### Logistic regression models for heifer pregnancy

To assess the association between body composition measurements and heifer pregnancy, two models were evaluated: a logistic regression (M1) and a multinomial logistic regression (M2). It should be acknowledged that these results represent a comparison between two developmental programs in which heifers were neither nutritionally restricted nor fed to extreme excess; therefore, these results should not be extrapolated to nutritional extremes.

Significant effects in the M1 were assessed using a Type III ANOVA, and results identified that FAT and treatment group were significant (*P* < 0.05) effects in the model. Although IMF was retained in the best-fit model by stepwise AIC, its effect was not statistically significant (*P* = 0.10). The M1 log-odds estimates and significance levels for treatment group, FAT, and IMF are presented in Table 9. Estimates for treatment group and FAT were significantly (*P* < 0.05) different from zero, indicating these predictors have a significant, non-zero effect, on pregnancy status in M1. The exponential of the log-odds estimates represents the odds ratio, which describes how the odds of pregnancy differ between levels of a categorical predictor or how the odds change with each unit increase in a continuous variable. Based on exponentiated M1 estimates, the odds of pregnancy in the restricted group were 5.05 times higher (*P* < 0.05) than in the non-restricted group, holding FAT and IMF constant at the average value. This demonstrates that when heifers from both groups were at the same level of FAT, heifers in the restricted group had higher odds of pregnancy compared to those in the non-restricted group. Across both treatment groups, for each 1 mm increase in FAT thickness, the odds of pregnancy increased by a factor of 1.92 (*P* < 0.05). While the odd ratio does not depend on the value of the predictor, the odds and probability of pregnancy for each group vary depending on the value of the predictor. For instance, the probability of pregnancy for a treatment group will vary depending on the level of FAT being considered. Table 10 presents the probability of a successful pregnancy outcome and 95% confidence interval at three levels of FAT thickness, split by treatment group. Across both treatment groups, the probability of pregnancy increases as the FAT level increases, with this trend exhibited at a greater magnitude in the non-restricted group. The estimated probability of pregnancy at 3 mm of FAT thickness for the non-restricted group exhibited a large confidence interval, likely due to the limited number of animals with low FAT thickness within this group.

**Table 9:** Estimated logistic regression coefficients, standard errors (SE), and significance levels (*P*-value) for heifer pregnancy.

Term	Coefficient	SE	*P*-value
**Intercept**	0.975	0.923	0.291
**Group^1^**	−1.622	0.813	0.046
**FAT^2^**	0.653	0.243	0.007
**IMF^3^**	−0.312	0.189	0.099

1 Group: Fixed effect for treatment group, with the coefficient representing the non-restricted group

2 FAT: fixed effect for backfat, scaled to millimeters.

3 IMF: fixed effect for percentage of intramuscular fat.

**Table 10:** Estimated probability of successful pregnancy outcome from logistic regression of heifer pregnancy at three levels of backfat thickness (FAT) in two treatment groups (restricted vs non-restricted).

FAT, mm	Group	Probability of pregnancy^1^	Asymptotic 95% confidence interval	
**3**	Restricted^2^	0.790	0.624	0.894
	Non-restricted^3^	0.425	0.149	0.758
**5.2**	Restricted^2^	0.940	0.822	0.982
	Non-restricted^3^	0.757	0.587	0.872
**7.5**	Restricted^2^	0.986	0.889	0.998
	Non-restricted^3^	0.933	0.808	0.979

1 Probability of pregnancy: probability of pregnant diagnosis at final pregnancy determination.

2 Restricted: Heifers developed to approximately 55% of expected mature body weight at the time of breeding.

3 Non-restricted: Heifers developed to approximately 65% of expected mature body weight at the time of breeding.

The results of the type III ANOVA for M2 identified FAT as a significant effect in the model (*P* < 0.05), and the effect of treatment group trended toward significance (*P* = 0.089). The M2 log-odds estimates and significance levels for treatment group and FAT are reported in Table 11. The outcome column indicates whether the coefficient reported compares the outcome of AI or NS to open heifers and the term for group is the contrast of the non-restricted to the restricted. The log-odds estimate comparing treatment group and NS bred is significantly (*P* < 0.05) different from zero, as are the both the AI and NS bred log-odds estimates for FAT (*P* < 0.05). The odds ratio, calculated by the exponential of the log-odds, reveal that holding FAT at the average. heifers in the restricted group had 3.80 times greater odds of AI pregnancy and 5.99 times greater odds of NS pregnancy compared to those in the non-restricted group. The AI and NS odds ratio for FAT indicate how the odds change with each 1 mm increase in FAT thickness. Accounting for treatment group, every 1 mm increase in FAT resulted in the odds of an AI pregnancy increased by a factor of 1.74, and the odds of a NS pregnancy increased by a factor of 1.55. The within-group probability of each pregnancy outcome at three levels of FAT thickness (3.0 mm, 5.2 mm, and 7.5 mm) and 95% confidence interval were reported in Table 12. The selected FAT levels represent the 25th, 50th, and 75th percentiles of the FAT measurements across both treatment groups. As FAT levels increased, the probability of being open outcome decreased in both treatment groups. In the restricted group, the probability of AI bred increased with FAT thickness, and the probability of NS bred remained relatively constant. In the non-restricted group, the probability of both AI-bred and NS-bred increased with FAT.

**Table 11:** Estimated multinomial logistic regression coefficients, SEs, and significance level for heifer pregnancy.

Term	Outcome^1^	Coefficient	SE	*P*-value
**Intercept^2^**	AI	**−**1.152	0.818	0.159
	NS	**−**0.165	0.776	0.832
**Group^3^**	AI	**−**1.346	0.858	0.117
	NS	**−**1.793	0.843	0.033
**FAT**	AI	0.555	0.220	0.012
	NS	0.439	0.217	0.043

1 Outcome: Level of pregnancy response outcome. Artificial insemination bred (AI) or natural service bred (NS) are both compared to open.

2 Regression coefficient estimate for fixed effects.

3 Group: Fixed effect for treatment group, with the coefficient representing the non-restricted group.

**Table 12:** Estimated probability (95% confidence interval) of three levels of pregnancy outcome (Open: not pregnant; AI: artificial insemination pregnant; NS: natural service pregnant) from multinomial regression of heifer pregnancy across three levels of backfat thickness (FAT) in two treatment groups of heifers (restricted vs non-restricted).

		Restricted^1^	Non-restricted^2^
		
FAT,^3^ mm	Outcome	Probability	
**3.0**	Open	0.17 (0.04, 0.31)	0.51 (0.09, 0.93)
	AI	0.29 (0.13, 0.44)	0.22 (**−**0.04, 0.48)
	NS	0.54 (0.37, 0.72)	0.27 (**−**0.03, 0.56)
**5.2**	Open	0.07 (**−**0.03, 0.16)	0.26 (0.08, 0.44)
	AI	0.38 (0.17, 0.58)	0.38 (0.19, 0.57)
	NS	0.56 (0.35, 0.77)	0.36 (0.17, 0.55)
**7.5**	Open	0.02 (**−**0.03, 0.08)	0.10 (**−**0.01, 0.21)
	AI	0.46 (0.11, 0.81)	0.53 (0.35, 0.70)
	NS	0.52 (0.17, 0.87)	0.38 (0.21, 0.55)

1 Restricted: Heifers developed to approximately 55% of expected mature body weight at the time of breeding.

2 Non-restricted: Heifers developed to approximately 65% of expected mature body weight at the time of breeding

3 FAT levels represent to the 25th, 50th, and 75th percentiles of the FAT measurements across both treatment groups.

**Figure 2:**
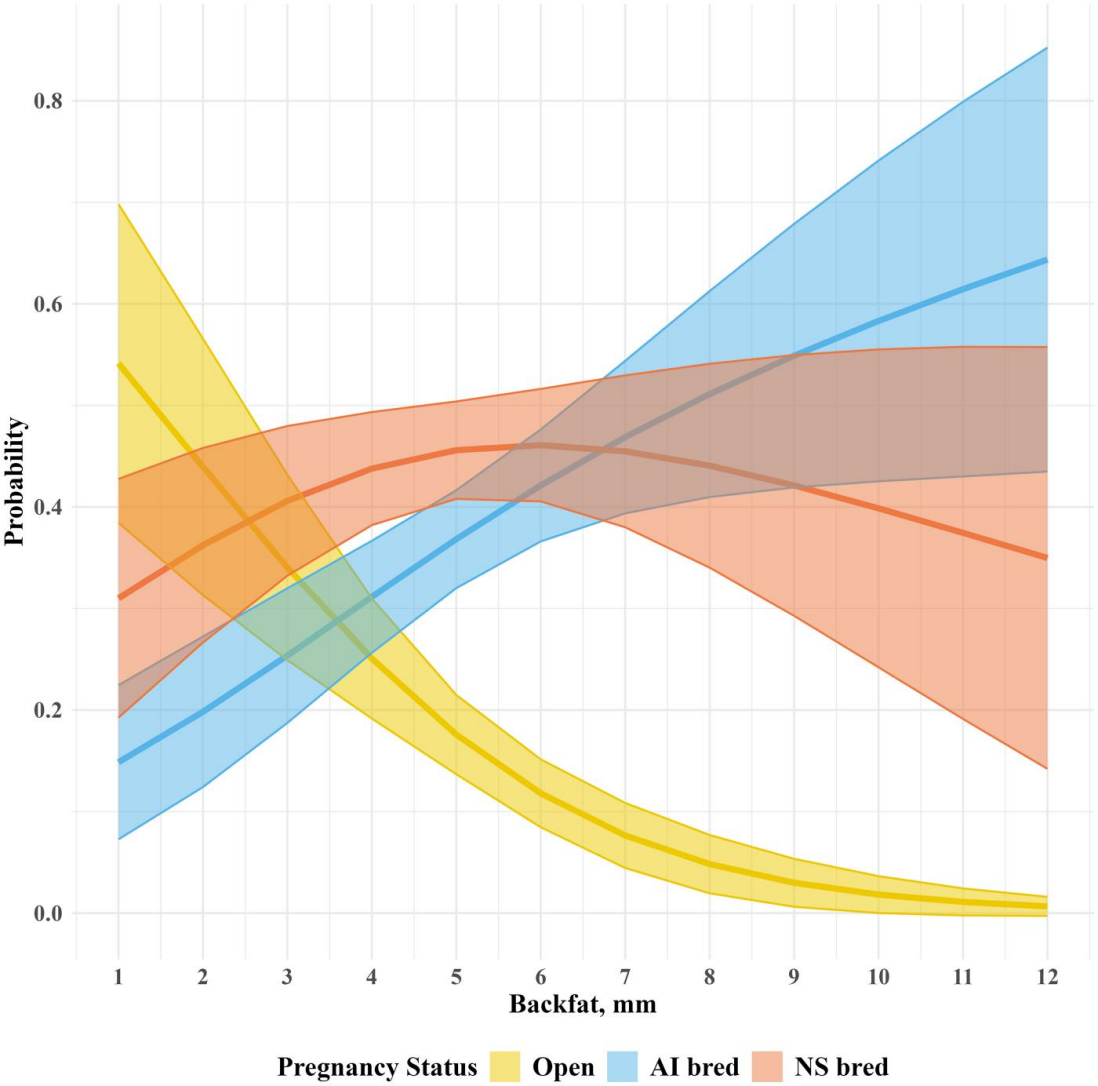
Probability of three levels of pregnancy outcome (Open; 0, AI bred; 1, and NS bred; 2) and 95% confidence interval (from multinomial regression) across the dataset range of FAT thickness (1–12 mm) averaged across the two treatment groups defined by developing heifers to either 55% of expected mature body weight at breeding (restricted) or to 65% of expected mature body weight at breeding (non-restricted).

Figure 2 highlights the change in pregnancy probability as FAT thickness increases, accounting for both treatment groups. The range of FAT thickness used in this figure represents the dataset range of FAT thickness for both treatment groups. The highest probability of being open occurs at 1 mm and 2 mm of FAT thickness. At 3 mm of FAT thickness through 6 mm, NS bred becomes the most probable outcome. Finally, at 7 mm of FAT thickness, AI bred was the highest probable outcome and continued to increase through 12 mm of FAT.

## Discussion

Heifers in the non-restricted group, developed to reach 65% of expected mature BW at breeding, had heavier BWs and larger body composition measurements during development compared to heifers in the restricted group, developed to reach 55% of expected mature BW at AI breeding. At the time of estrus synchronization, ten days prior to AI breeding, the average BW was 339.68 kg in the restricted group and 396.62 kg in the non-restricted group, with the mean values between treatment groups being significantly (*P* < 0.05) different. Similar research that compared heifers that were developed on a winter grazing system to heifers developed in a dry lot found that heifers developed in the dry lot had significantly higher (*P = 0.02*) BWs at breeding (Funston and Larson 2011).

High Spearman rank correlation coefficients for BW and REA indicated a monotonic relationship between heifers within treatment group during a given timeframe. Slightly lower rank correlations for FAT and RUMP were observed relative to BW and REA during the same timeframe. This suggests there were slightly more differences in the development of FAT and RUMP in pre-breeding heifers compared to BW and REA. This agrees with what is known about the growth rate of muscle and fat cells. All hyperplasia of muscle cells occurs before birth, while hyperplasia of fat cells continues during the pre-pubertal stage (Du et al. 2013). Thus, the greater reranking of FAT and RUMP may result from continuous fat cell deposition during this time of development, whereas REA muscle cells are strictly undergoing hypertrophy. Results from both logistic regression models indicated that FAT was a significant predictor of pregnancy outcome. Therefore, the timing and development of FAT may be an important influence of pregnancy status.

Moderate rank correlations for IMF were observed between day 0 and 55 in both treatment groups, indicating notable differences in the development of IMF for early post-weaning heifers implying that the heifers with high IMF values shortly after weaning (day 0) did not consistently have high values for IMF at day 55. As heifers became older and closer to the time of AI breeding, rank correlations increased, suggesting that differences in IMF development decreased with age between heifers within treatment groups. Biologically, the Spearman’s rank correlation for IMF are logical. It is understood that IMF cell hyperplasia begins in the late fetal stage and continues through post-parturition until approximately 250 days of age (Du et al. 2013). At day 0, the average age of heifers was 180 days old, indicating that these females would have been still depositing IMF cells. This could explain the considerable amount of re-ranking between day 0 and day 55 in both treatment groups for IMF measurements. After day 55, Spearman rank correlations for IMF increased in both treatment groups, suggesting a reduced re-ranking of heifers as development progressed. Throughout growth and development, IMF has been shown to uniformly increase (Bruns et al. 2004; Albrecht et al. 2006; Ahn et al. 2023). This may explain the considerable increase in IMF Spearman rank correlations after day 55, as the degree of re-ranking gradually declined compared to the comparison from day 0 to day 55. In a study by Evans et al. (2004), it was noted that prepubertal heifers have less IMF compared to cycling heifers that became pregnant. However, results from the logistic regression performed in the current study indicate that IMF was not a significant predictor of pregnancy outcome.

Pre-breeding performance was evaluated by assessing RTS, pelvic area, and estrous response to synchronization. To evaluate reproductive tract maturity, RTS are assigned 4 to 6 wk prior to breeding and assess the uterine horn tone and dimension, ovary size, and ovarian structures (Andersen et al. 1991). Heifers scoring a 4 or 5 are assumed to have reached puberty onset and are cycling (Moorey and Biase 2020). On average, both treatment groups scored a 4 for RTS, suggesting that most heifers in this study should have been cycling at breeding. No studies have reported the effect of nutrition on RTS, but Andersen et al. (1991) suggested one application of RTS could be to indicate nutritional status and the nutritional needs of heifers when performed early enough to make nutritional adjustments prior to breeding.

Pelvic area measurements in yearling heifers can be used as a culling tool to reduce the risk of dystocia in a heifer. At yearling measurements, heifers should have a pelvic area of 150 cm^2^ or larger to be considered as a candidate for breeding (Patterson et al. 2013). A significant difference (*P* < 0.05) in the average pelvic area was observed between treatment groups, with the non-restricted group having a larger average pelvic area. However, the mean pelvic area of both groups was above the threshold of 150 cm^2^. These results agree with a previous study concluding that heifers on a higher gain diet have larger pelvic areas compared to heifers on moderate and lower gain diets, suggesting that diet can influence the development of the pelvic area (Fleck et al. 1980).

Findings from a previous study indicated that heifers developed to 65% of expected mature BW had a greater percentage of females pubertal prior to AI breeding compared to heifers developed to 55% of expected mature BW (Funston and Larson 2011). The association between the nutritional status of heifers and the earlier onset of puberty is due to how nutrients are partitioned in the body. Nutrients are first allocated to support basic growth, physical activity, and energy storage, followed by pregnancy, lactation needs, and additional energy reserves, then finally the estrous cycle and the initiation of pregnancy (Short and Adams 1988). Heifers developed to higher percentages of expected mature BW are likely to have additional nutrients earlier to be partitioned to support the estrous cycle and initiation of pregnancy compared to heifers developed to lower expected percentages of mature BW. Biologically, increased nutritional status has been associated with an earlier onset of the LH pulses that lead to the LH surge shortly before puberty onset ( Short and Adams 1988; Gasser et al. 2006; Day 2015). However, puberty has been demonstrated to be multifactorial, as heifers on a higher gain diet reached puberty earlier compared to heifers on a lower gain diet, but were heavier at puberty (Short and Bellows 1971). Overall pregnancy rates (AI and NS combined) have been shown to be greater for heifers that have undergone puberty onset prior to breeding compared to prepubertal heifers (Roberts et al. 2017). Therefore, it is important to ensure puberty onset occurs prior to breeding.

Puberty onset was not directly measured in this study; however, response to the synchronization protocol was recorded to identify the level of estrus expression at the time of AI breeding. A previous study developed a 3 level PS system where each score corresponded to the following observation: 1 less than 35% abrasion, 2 between 35% to 65% abraded, and 3 over 65% abraded (Walker et al. 2014). The current study followed this scoring system and found that heifers developed to a higher percentage of mature BW at breeding had a statistically (*P *< 0.05) greater number of PS 2 and 3 compared to heifers developed to lower BWs at breeding. Therefore, heifers in the non-restricted group had a greater response to estrus synchronization compared to heifers in the restricted group. Another study developed a PS system on a scale of 0 to 4 with corresponding scores as follows: 0 lost patch; 1 less than 25% abraded; 2 less than 50% abraded, 3 less than 75% abraded; and 4, over 75% abraded (Pohler et al. 2016). In this study, it was found that heifers with higher PS at timed-AI breeding had higher concentrations of bovine pregnancy-associated glycoprotein (bPAG) on day 28 of gestation, indicating a higher pregnancy success rate in heifers with higher PS (Pohler et al. 2016). Other research has suggested that successful pregnancy rates increase when heat detection patches have been abraded by 50% or greater (Oliveira Filho et al. 2019).

After AI breeding, all heifers were managed as one group on pasture, and carcass measurements were taken at final pregnancy diagnosis to evaluate the change in body composition measurements due to pasture management. On average, heifers in both groups lost REA, FAT, and RUMP, with this decrease being more prominent in the non-restricted group. Interestingly, heifers in both groups, on average, gained IMF during pasture management. Limited research is available regarding changes in body composition due to the transition from dry lot to pasture management. A previous study observed that heifers fed a lower-gain pre-breeding diet had lighter BWs at breeding compared to heifers fed a higher-gain diet (Funston and Larson 2011). However, lower-gain heifers did have compensatory gain on pasture and had similar pre-calving weights. Similarly, another study reported pasture-raised heifers had a higher average daily gain compared to dry lot heifers during gestation (Mulliniks et al. 2013).

In the current study, four AI pregnancies were lost in the restricted group and two in the non-restricted group. Previous work investigating heifer pregnancy loss during the late embryonic and early fetal stages has reported losses ranging from 1.5% to 4.4% (Perry et al. 2007; Richardson et al. 2010). A meta-analysis evaluating this stage of pregnancy loss in both *Bos indicus* and *Bos taurus* cattle found an average loss of 8.2% in beef heifers and 5.1% in cows (Reese et al. 2020). As a percentage, AI pregnancy losses were higher in our study compared to previous work, with a 19.0% and 6.9% pregnancy loss in the restricted and non-restricted groups, respectively. This higher loss experienced could be due to the abrupt shift to pasture management after AI breeding. A drastic shift in post-breeding management has been shown to influence pregnancy loss. Heifers administered a lower-gain diet lost more pregnancies after transitioning to summer pasture management compared to heifers administered a higher-gain diet (Short and Bellows 1971). This study demonstrated how abruptly switching from drylot to pasture management could impact AI pregnancy rates.

Numerically, more heifers in the non-restricted group were AI-bred, while more heifers in the restricted group were NS-bred. However, no difference in final pregnancy rates was observed between heifers in the restricted group compared to the non-restricted group following a 45-day breeding season. Previous findings are consistent with the current study, as an increase in AI pregnancy rates was observed for heifers developed to 65% of expected mature BW at breeding compared to 55% of expected mature BW, but this difference was not significant (Funston and Larson 2011). Furthermore, final pregnancy rates did not differ between heifers developed to 65% of expected mature BW at breeding compared to 55% of expected mature BW. A study comparing NS pregnancy rates found no difference in pregnancy rates between heifers administered a high-gain or low-gain diet. Conversely, other literature had reported pasture-developed heifers had higher pregnancy rates than dry lot-developed heifers (Mulliniks et al. 2013). These results suggest that heifers developed to a higher percentage of mature weight by feeding a higher ADG diet may conceive earlier compared to heifers at a lower percentage of mature BW.

It is important to note that while final pregnancy rates did not differ between treatment groups, more heifers in the restricted group were culled due to low RTS and small pelvic areas compared to heifers in the non-restricted group prior to breeding. Of the total number of heifers retained as replacements, 11% of the heifers in the restricted group were culled and 6% in the non-restricted group. Developing heifers to a lower percentage of BW at the time of breeding may lead to the unintended consequences of increased culling prior to breeding due to inadequate nutrition, which would not be reflected in the final pregnancy rates, as pregnancy rates are expressed as the percentage of pregnant animals over the total number of animals exposed. If a heifer is culled due to failing to meet minimal requirements for breeding, this would not be expressed in final pregnancy percentages, as the heifer was never exposed to a bull. Based on the number of initial heifers in each group, more heifers in the non-restricted group made it to exposure and were bred compared to the heifers in the restricted group. This study indicates that heifers developed to lower levels of BW result in higher levels of culling and a lower number of heifers exposed, thus an increased economic loss due to lost investment in replacements, relative to heifers developed to higher BWs.

Fat deposition influences fertility in beef cows (Wettemann et al. 2003; Zieba et al. 2005). However, its impact does not appear to be linear. Both under-conditioned and over-conditioned females experience lower fertility (Wettemann et al. 2003; Martínez et al. 2021). It is important to note that heifers in this study were not severely underconditioned or over conditioned. Results are intended to compare pregnancy response and body composition measurements of two developmental strategies. These results should not be extrapolated to heifers that are nutritionally deprived during development or obese heifers.

When considering FAT using logistic regression analysis, results from both heifer pregnancy models (M1 and M2) indicate that the treatment group and backfat thickness were significant predictors of pregnancy response. While research associating actual FAT measurements and pregnancy outcome is limited, a comprehensive review was published regarding a multi-study effort in Australia to better understand the relationship between feedlot and carcass traits with maternal performance (Pitchford et al. 2017). This project was inspired by the reservations Australian cattle producers had regarding the implications of increased feedlot and carcass performance on maternal performance. The first study detailed in this project was industry-focused and evaluated body composition in females before and after calving during their first two parities, with the aim of improving understanding of the female body compositional changes that occur from supporting a calf and aging. The second study used research lines selected for either high or low FAT breeding values to evaluate subsequent reproductive performance. Outcomes from the industry study reported high genetic correlations between ultrasound carcass measurements taken before calving and after weaning for first- and second-calf females and also between all measurements taken over the first two parities (Donoghue et al. 2017). The authors concluded that there is a genetic component to body composition for breeding females measured before calving and after weaning, and improvements in body composition can be made through selection. Furthermore, selection for increased body composition did not appear to hinder maternal performance, as heifers with high FAT breeding value had higher pregnancy rates compared to heifers with low FAT breeding value (Jones et al. 2017). Therefore, the findings from the comprehensive maternal project support the conclusions of the current study that suggest backfat plays a critical role in heifer pregnancy.

In M1, the best-fit model based on AIC included IMF in the model; however, IMF was not a significant (*P* = 0.10) predictor. As previously mentioned, it was found that prepubertal heifers had lower percentages of IMF compared to pubertal heifers that became pregnant (Evans et al. 2004). The results from the current study would suggest that IMF may not influence pregnancy outcomes. Final pregnancy status also appeared to be independent of REA. A study examining the effects of pregnancy on body composition measurements found that open heifers had statistically larger REA than pregnant heifers and numerical differences in fat content, with the pregnant heifers having more FAT and percent kidney pelvic heart fat than open heifers (Bishop et al. 2003). However, no difference in marbling scores was observed between the open and pregnant heifers (Bishop et al. 2003). Further research is needed to understand the exact effects of muscle and the selection for improved muscle trait performance. Results from this study emphasize the need for operation-specific heifer development practices, as there are benefits and consequences to developing heifers to either 55% or 65% of mature BW at breeding.

##  

In conclusion, this study evaluated the impact of phenotypic ultrasound-measured carcass traits on the reproductive performance of heifers developed to different expected mature BWs at breeding. Heifers developed to a higher expected mature BW had higher body composition measurements during development, large pelvic areas, higher response to estrous synchronization, and numerically more heifers bred via AI compared to heifers developed to a lower percentage of expected mature BW at breeding. Heifers developed to higher BWs also had less culling prior to AI breeding due to low RTS and pelvic areas. Final pregnancy rates after culling did not differ between the two treatment groups, and heifers in the restricted group continued to gain BW and lost body composition at a lower magnitude than heifers in the non-restricted group when management transitioned to pasture.

Backfat and treatment group were significant predictors of heifer pregnancy outcome. Current practices use BW and BCS to evaluate puberty onset and reproductive maturity. Results from this study demonstrate the need for further research investigating the relationship between FAT thickness and heifer pregnancy status and the utility of FAT in selecting replacement heifers. As overall industry trends place downward selection pressure on FAT to improve yield grades at the time of harvest, the consequences of this pressure on heifer pregnancy should be considered.

## Abbreviations

AI: artificial insemination
ADG: average daily gain
BCS: body condition score
BW: body weight
CIDR: controlled internal drug release
FAT: backfat thickness
GnRH: gonadotropin-releasing hormone
IMF: intramuscular fat
NS: natural service
PS: Patch Score
REA: ribeye area
RTS: reproductive tract score
RUMP: rump fat thickness
